# Diode Laser-Assisted Pulp Polyp Excision and Canal Disinfection in a Primary Molar: A Case Report

**DOI:** 10.7759/cureus.54315

**Published:** 2024-02-16

**Authors:** Himani Parakh, Nilima R Thosar, Aakriti Chandra, Neha Pankey

**Affiliations:** 1 Pediatric and Preventive Dentistry, Sharad Pawar Dental College and Hospital, Datta Meghe Institute of Higher Education and Research, Wardha, IND

**Keywords:** diode laser, pediatric dentistry, primary molar, canal disinfection, pulp polyp

## Abstract

This case report presents the successful application of diode laser technology in the management of a pulp polyp in a primary molar, showcasing its efficacy in both excision of the proliferative tissue and subsequent canal disinfection. An eight-year-old patient with a symptomatic primary molar exhibiting a pulp polyp was selected for this intervention. The diode laser, with its specific wavelength and precise tissue interaction, allowed for a minimally invasive and efficient removal of the pulp polyp. Additionally, the laser was utilized for thorough disinfection of the root canals, targeting bacterial pathogens while preserving surrounding healthy tissue. The case was monitored over a three-month follow-up period, demonstrating favorable clinical and radiographic outcomes. This report emphasizes the potential of diode laser technology as a feasible adjunct in the treatment of pulp polyps in primary molars, showcasing its benefits in terms of reduced invasiveness, enhanced precision, and effective canal disinfection in pediatric endodontics.

## Introduction

Pulp polyp, also referred to as chronic hyperplastic pulpitis or proliferative pulpitis, refers to a tissue condition characterized by excessive tissue growth in the pulp chamber of the tooth. This condition usually occurs in severely decayed teeth, fractured teeth, or teeth with deep cavities, especially in young children or among those people who have a high immune response [1]. The growth manifests as a reddish, proliferative mass that is frequently discernible on the molar occlusal (chewing) surface due to its abundant blood supply. It is a reaction to long-term, decay-induced, chronic pulp irritation or infection. The body is trying to shield the tooth from additional harm or infection, which is why there is an excessive growth of tissue.

When a tooth is affected by a pulp polyp, it is not uncommon to observe signs of internal root resorption and a periapical lesion, also known as apical periodontitis. The presence of internal root resorption points to long-term inflammation and activity of odontoclasts, while the periapical lesion is a signal of severely inflamed pulps, such as irreversible pulpitis or an infected root canal system. Parents usually worry upon noticing a pulp polyp in their child’s mouth because of its distinctive clinical appearance, discomfort while having meals, as well as occasional bleeding if aggravated [2]. Pulp polyps can be treated by the removal of the pulp polyp followed by pulpectomy or root canal therapy, which removes the diseased pulp and seals the canals, or, in extreme situations, by extraction of teeth. Along with dental cleanliness and care, quickly treating cavities at the initial stages is crucial in halting the development of diseases like pulp polyps to this advanced level [3].

## Case presentation

**Figure 1:**
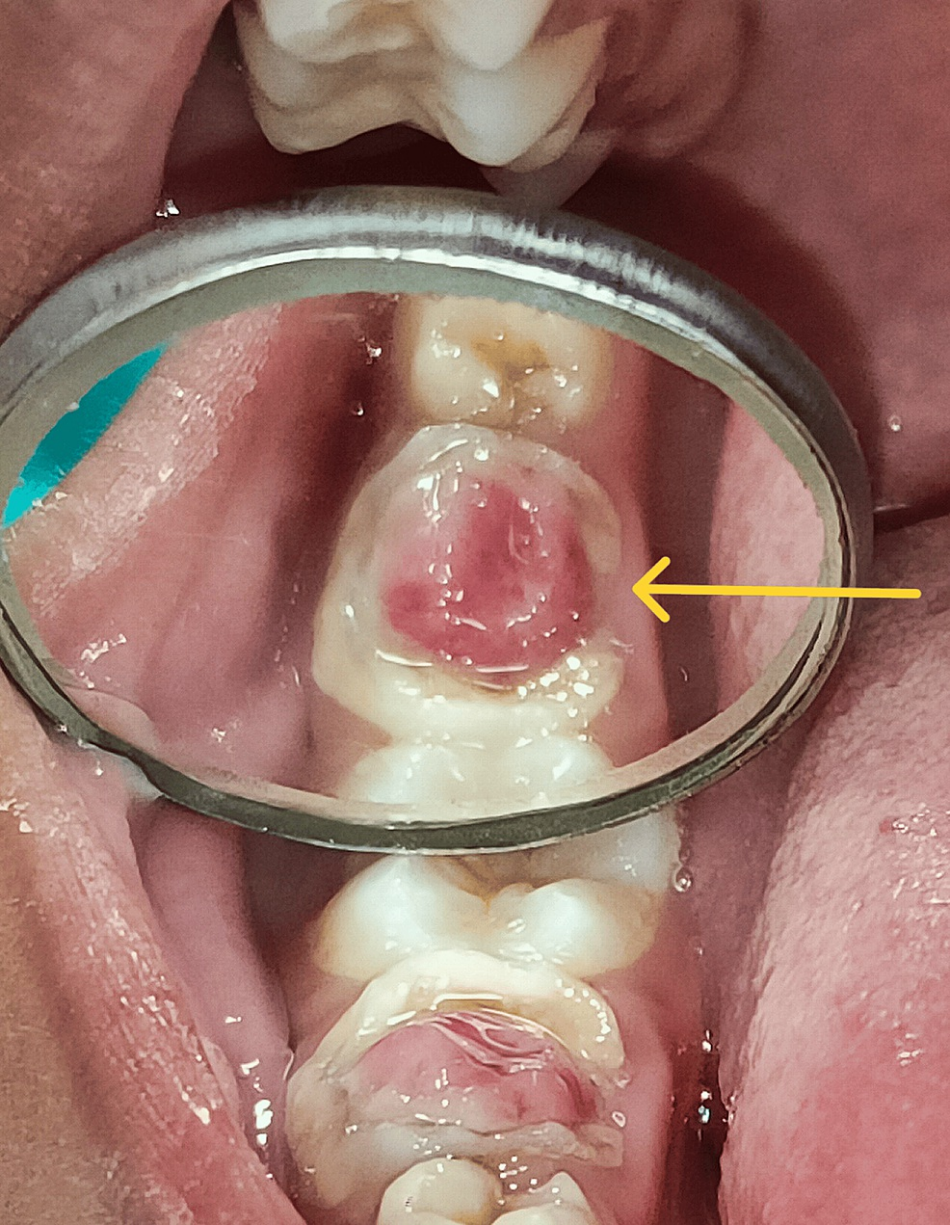
Pulp polyp (arrow) in mandibular primary second molar (85)

**Figure 2:**
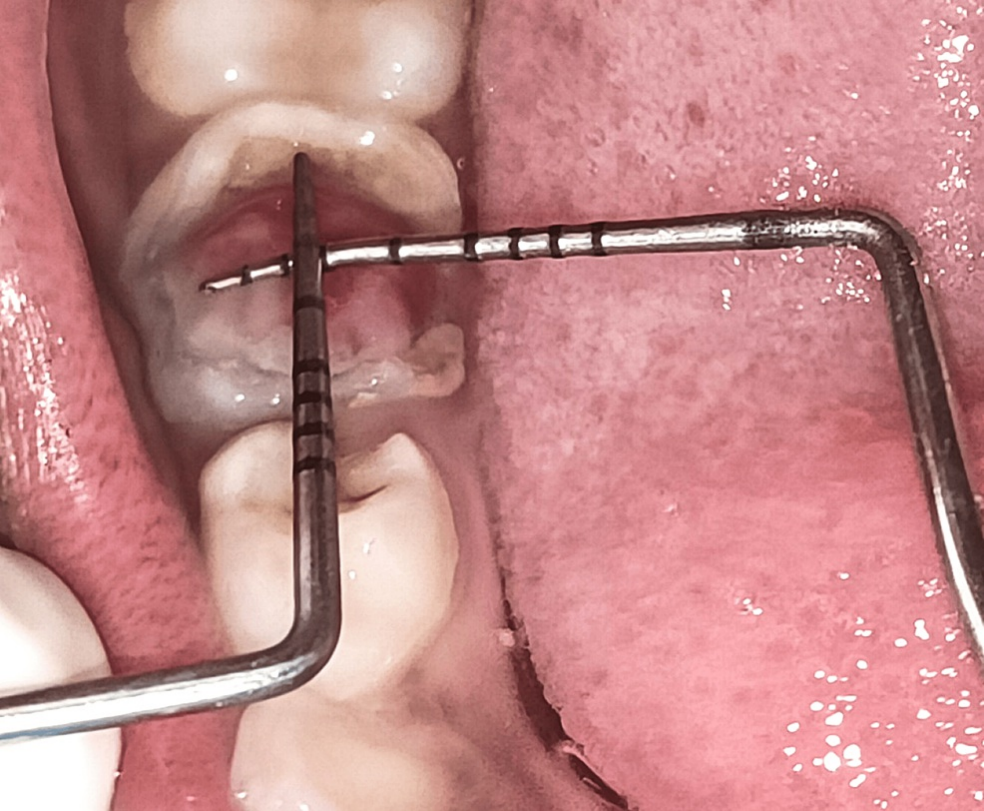
Dimensions of the pulp polyp (6 mm x 5 mm)

An eight-year-old female patient reported to the Department of Pediatric and Preventive Dentistry, whose parents complained of a decayed tooth and growth of mass at the lower right back tooth region of the jaw for the past three months, which resulted in disrupted mastication and was accompanied by mild, intermittent discomfort and bleeding while brushing. Based on the patient's medical history, it was determined that she did not have any systemic medical issues, allergies, or a history of using medications or recreational drugs. Additionally, there were no past surgical procedures on record. As a result, there was no need for the patient to be referred for a medical consultation. Upon intraoral examination, the exophytic mass (Figure 1) was causing problems with eating and closing the jaw. This led to intermittent pain associated with bleeding. The radiographic examination showed internal root resorption at the distal root of the primary right first mandibular molar associated with a large carious lesion on the same tooth. The patient was diagnosed with a pulp polyp of 6 mm x 5 mm mesiodistally to buccolingually (Figure 2).

Treatment

**Figure 3:**
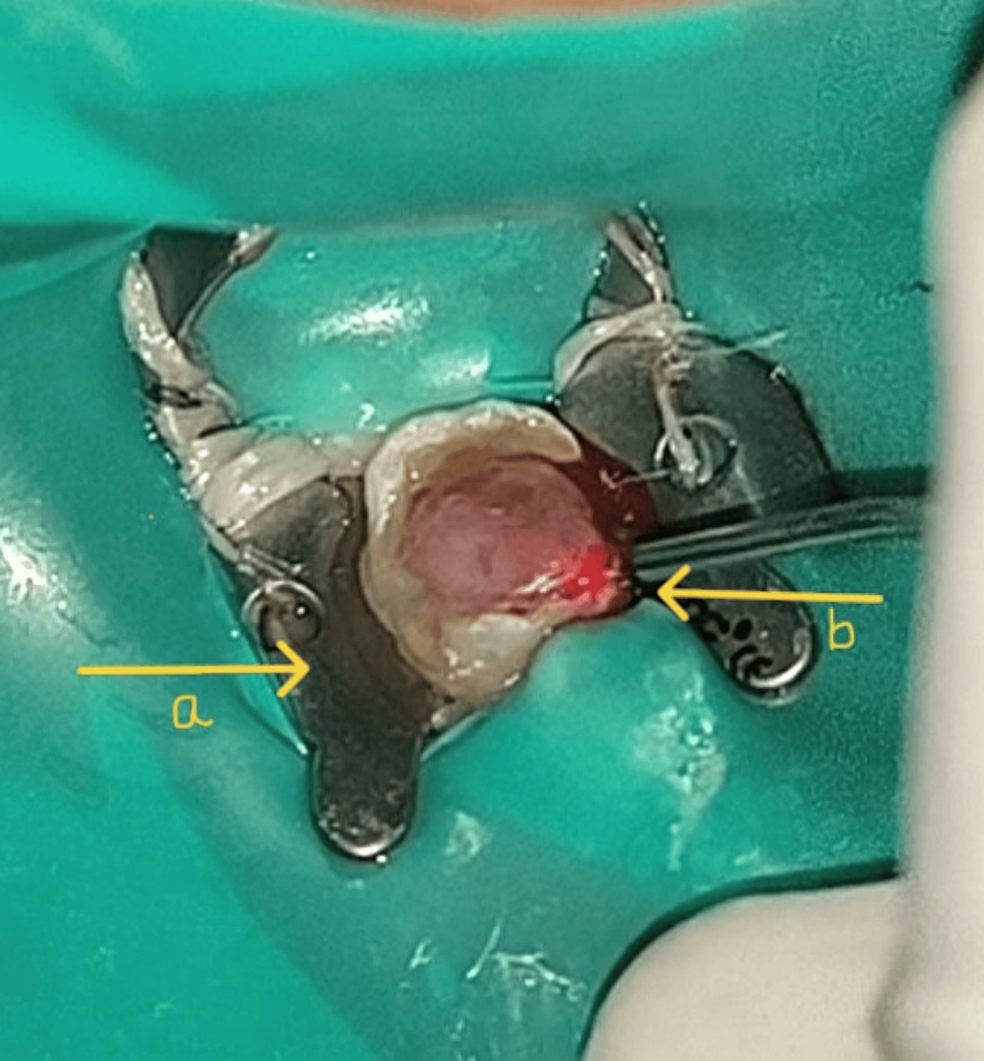
Rubber dam isolation and diode laser tip pointed toward the pulp polyp (a) Rubber dam isolation; (b) Initiated laser tip

**Figure 4:**
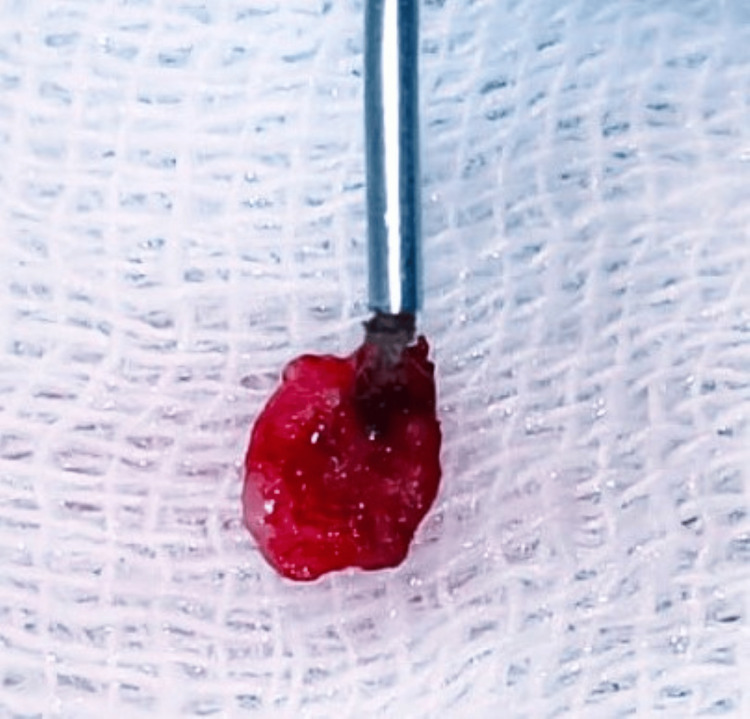
Excised pulp polyp

**Figure 5:**
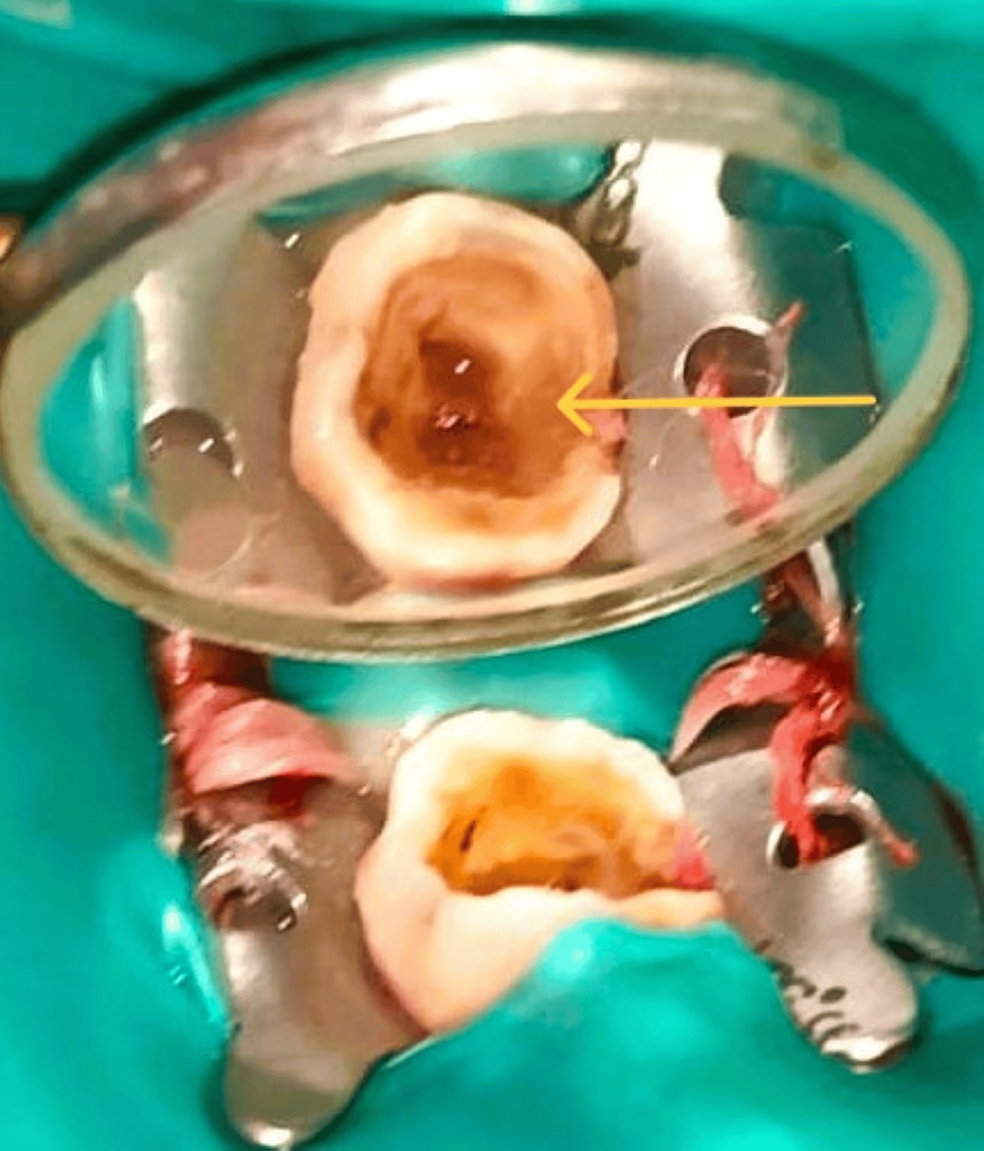
Disinfection and irrigation using 2.5% sodium hypochlorite solution

**Figure 6:**
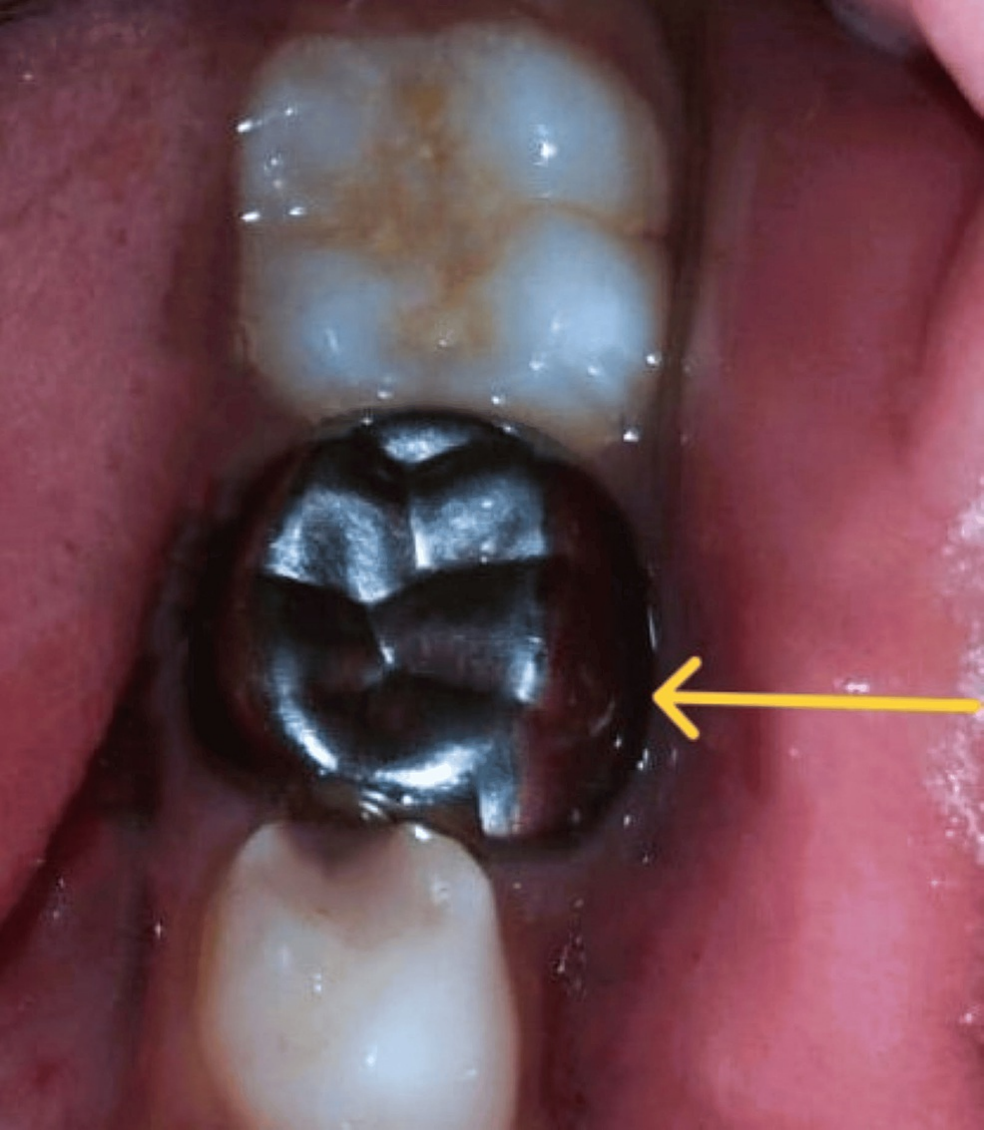
Placement of stainless steel crown (arrow) after completion of the endodontic treatment

Prior to the excision of the pulp polyp, blood investigations were done for the patient which were found to be within normal range, and parental consent was taken for the procedure to be performed under local anesthesia. After getting consent from the patient’s parent, the procedural area was anesthetized by injecting 2% lignocaine (with 1:80,000 adrenaline) into the inferior alveolar nerve; the nerve was effectively blocked using a dosage of 1.8 ml. Under complete rubber dam isolation using a high-power diode laser (EpicX, Biolase, USA) (Figure 3), the pulp polyp was eliminated (Figure 4), and then low-level diode laser radiation was used to clean the canal orifices after a cotton pellet was bathed in regular saline for five minutes. An initiated fiber with continuous wave and contact mode, wavelength of 940 nm, power of 1.4 W, and fiber length of 400 µ as the laser parameters was used for the pulp polyp removal. Using a power of 0.1 W at the same wavelength and fiber length, the root canal was further disinfected. After this procedure, complete pulp extirpation was done, and bio-mechanical preparation (BMP) up to no. 30 K-file was done. After complete BMP and thorough irrigation, a dry cotton pellet was used to dry the tooth, and the cavity was temporized using Cavit (3M ESPE, St. Paul, USA). In the next appointment, the temporary restoration was removed and then thorough irrigation using 2.5% sodium hypochlorite was done (Figure 5). The canal was dried using negative aspiration and paper points. The canal was obturated using zinc-oxide eugenol paste. An interim restorative therapy using glass ionomer cement (Fuji IX, GC Europe) was applied as per the manufacturer's instructions. The patient was recalled after one week to check for any post-operative complications. As no pain was experienced, the permanent restorative therapy was given using glass ionomer cement followed by the placement of a stainless steel crown with respect to 85 (Figure 6).

Follow-up

The patient was followed up after three months. Clinical observations as well as radiographic evaluation indicated a good treatment outcome, and the patient reported no pain.

## Discussion

Chronic hyperplastic pulpitis, also known as pulp polyp or proliferative pulpitis, is a reactive lesion of pulpal tissue that appears as a proliferative granulation development from the pulp, obstructing the carious defect with intact walls surrounding it. The pulp polyp's color can range from the granulation tissue's cherry red to the moist keratinized epithelium's opaque white, contingent on the length of time and the existence of dilated blood vessels.

Compared to permanent dentition, pulp polyp management is different. The course of therapy for deciduous teeth would be usually to extract them and place a space maintainer in their place until the underlying permanent teeth erupted [4]. But in this case, the tooth is being preserved as a natural tooth and acts as the best space maintainer in children.

With differing degrees of effectiveness, a variety of therapeutic techniques, including electrocautery and scalpels, have been tried to remove pulp polyps [5]. On the other hand, lasers offer a wide range of advantages, including a bloodless field, increased visibility, no need for sutures, shorter recovery time, and extremely low pain following surgery [6,7]. Dental offices across the country frequently employ diode lasers for various purposes. Solid-state elements, including gallium, arsenide, aluminum, and indium, are used in diode lasers, which are semiconductor lasers. Wavelengths ranging from 810 to 980 nm are offered for these [6,8]. We removed the pulp polyp, in this case, using a 940 nm diode laser. In contrast to other lasers that have a preference for water and hydroxyapatite, diode lasers are effective in coagulating because they have a preference for pigmented and vascular lesions that consist of hemoglobin and melanin, among other chromophores [8,9]. Through this case, the potential of a diode laser as a beneficial option for the excision of pulp polyps becomes apparent due to its capacity to decrease procedural duration and diminish post-operative discomfort.

When it comes to achieving good coagulation in human primary molars, Gupta et al. found that laser pulpotomy using high-power diode lasers performed better than both electrosurgery and ferric sulfate pulpotomy. This was evident in both clinical and radiographic results [10]. When Uloopi et al. used low-level diode lasers for pulpotomy, they observed that the results were similar to those of the mineral trioxide aggregate pulpotomy approach, suggesting that low-level laser therapy might be used for pulpotomy in primary teeth [11]. This is a three-month follow-up study to effectively treat a pulp polyp using a diode laser in a pediatric patient without the need to extract the tooth.

## Conclusions

In conclusion, the application of diode laser technology for pulp polyp excision and canal disinfection in a primary molar showcased promising results in terms of removal of the pulp polyp. Excellent coagulation was evident, thus preserving the natural tooth and reducing postoperative discomfort. Further research and long-term studies are necessary to establish its broader clinical implications and compare outcomes with traditional methods. This case report suggests that diode lasers could be a valuable addition to the armamentarium of pediatric endodontics, offering a potential alternative or adjunctive tool for pediatric dentists to manage pulp polyps in primary molars.
